# Effect of an enhanced recovery after surgery program on total hip and knee arthroplasty in a university hospital: a two-cohort study

**DOI:** 10.1007/s00264-026-06744-z

**Published:** 2026-02-02

**Authors:** Eric Albrecht, Alexandre Koninckx, Corey Kull, Marcio Oliveira, Valérie Addor, Jean-Benoit Rossel, Julien Wegrzyn

**Affiliations:** 1 University Hospital of Lausanne, Lausanne, Switzerland; 2 University of Lausanne, Lausanne, Switzerland

**Keywords:** Hip arthroplasty, Knee arthroplasty, ERAS, Analgesia, Clinical pathway

## Abstract

**Background:**

Enhanced Recovery After Surgery (ERAS®) is a coordinated, evidence-based program delivered through a multidisciplinary team, which objective is to improve outcomes and patient satisfaction, while minimizing complications. The objective of this two-cohort study is to evaluate the clinical impact of an ERAS program on elective primary hip (THA) and knee (TKA) arthroplasties with regards to hospital length of stay, during the first 12 months after implementation.

**Methods:**

We compared a retrospective pre-ERAS with a prospective ERAS cohort. Key aspects of this program included preoperative education, minimal fasting, standardised, anaesthetic and surgical techniques, multimodal analgesia, and early mobilization. The primary outcome was hospital length of stay. Other outcomes included rest, dynamic pain scores, and rates of complications.

**Results:**

From December 1st, 2021 to November 30th, 2022, data from 267 patients (138 THA, 129 TKA) were compared with data from 258 patients (128 THA, 130 TKA) collected between December 1st, 2022, and November 30th, 2023, who underwent the ERAS® program (total: 525 patients). The mean hospital length of stay for THA patients before ERAS® was 5.5 ± 2.9 days versus 4.5 ± 2.0 days after ERAS® implementation (*p* = 0.002). For TKA patients, it was 6.6 ± 3.1 days before vs 5.6 ± 1.9 days after ERAS® implementation (*p* = 0.001). Rest, dynamic pain scores, and rates of complications were similar between groups except for pneumonia in patients undergoing TKA.

**Conclusion:**

The implementation of an ERAS® program for hip and knee arthroplasty led to a reduced hospital length of stay, below the Swiss national average, without impacting pain outcomes and rates of complications.

## Introduction

Based on evidence and standardized care across the surgical timeline, Enhanced Recovery After Surgery (ERAS®) is a coordinated, evidence-based program delivered through a multidisciplinary team [1], whose objective is to improve patient-centred outcomes and satisfaction, while minimizing postoperative complications, which ultimately lead to a reduction in hospital length of stay. This has been demonstrated specifically for lower limb arthroplasty by different centres [2–5]. Key elements of an ERAS program are preoperative education and optimisation, reduced fasting time, standardised analgesic, anaesthetic and surgical techniques, and early postoperative care [6].

The objective of this two-cohort study is to evaluate the clinical impact of an ERAS program on elective primary hip and knee arthroplasties with regards to hospital length of stay, pain outcomes, rates of complications and 30-day re-admission rates, during the first 12 months after implementation.

## Patients and methods

### Study design and setting

We conducted a single-centre, two-cohort study in a high-volume arthroplasty unit of a University Hospital. This protocol adhered to the principles described in the Declaration of Helsinki and received ethics approval from the institutional review board (reference 2024–01628). A prospective cohort of adult patients scheduled for primary elective total hip or total knee arthroplasty during the first 12 months after the implementation of our ERAS® program (December 1 st, 2022 – November 30th, 2023) was compared with a similar retrospective control cohort of patients treated under the previous conventional pathway during the 12 months immediately preceding December 1 st, 2022. Eligible patients were adults over 18 years old who underwent elective total hip or knee arthroplasty. Exclusion criteria were: revision procedures, simultaneous bilateral arthroplasty, fracture or tumour pathology, and refusal of data usage. All patients included signed the general consent waiver.

### ERAS® program

Based on the comprehensive set of recommendations previously published by Wainwright et al specifically for THA and TKA [6], we have elaborated our own approved ERAS program entirely based on the most contemporary literature. This ERAS program, containing 18 evidence-based items grouped into pre-operative, intra-operative and post-operative domains is summarised in Table 1. In brief, the key elements are: pre-operative optimisation; evidence-based fasting; standardised anaesthesia and analgesia (preferential use of spinal anaesthesia with low dose of intrathecal morphine, multimodal analgesia); blood-sparing strategies; surgical considerations (minimally invasive approaches where feasible, avoidance of routine drains, early removal of urinary catheters if inserted); early postoperative care; and complication prophylaxis.

**Table 1:** Total hip (THA) and knee (TKA) arthroplasty pathway

*Pre-operative (7 items)*
• Structured patient education program and take–home booklet: counselling, informing patients about the procedure, recovery timeline, and self-care responsibilities
• Risk stratification and medical optimization, including management of comorbidities
• Anaemia screening and correction (intravenous iron ± erythropoietin if hemoglobin < 130 g.L⁻^1^)
• Venous thrombo-embolism risk stratification and plan
• Kondrup Nutritional Risk Screening – 2002 (≥ 3 triggers dietitian referral)
• Prehabilitation (exercise and nutritional support to build strength and stamina)
• Preoperative fasting: solids up to 6 h and clear fluids up to 2 h before induction, with routine carbohydrate loading (200 mL 12.5%) up to 2 h preoperatively
*Intra-operative (10 items)*
• Standardised regional anaesthesia: no premedication, spinal anaesthesia with bupivacaine and low-dose intrathecal morphine (100 µg), along with peripheral nerve block (adductor canal and selective tibial nerve blocks for patients undergoing TKA; no blocks for patients undergoing THA)
• Opioid-sparing multimodal analgesia: intravenous dexamethasone (0.1–0.2 mg.kg^−1^), paracetamol (1 g), ketorolac (30 mg), magnesium (40–50 mg.kg^−1^)
• Postoperative nausea and vomiting prophylaxis: dexamethasone, ondansetron
• Light sedation with propofol on request
• Normothermia maintenance: forced-air warming system and warmed fluids
• Blood-sparing strategies: single-dose intravenous tranexamic acid (15 mg.kg⁻^1^) before incision, restrictive transfusion thresholds, and meticulous haemostasis
• Minimally invasive approach with standardisation of the surgical workflow and robotisation of the procedures for total knee arthroplasty, without tourniquet and surgical drains, to reduce soft tissue trauma
• Perioperative fluid management: restrictive fluid administration and maintenance of euvolemia
• No routine urinary catheter
• Standardised antibiotic prophylaxis (cefuroxime 1.5 g 30–60 min before incision)
*Post-operative (7 items)*
• Early return to oral fluid intake and normal diet, and early mobilisation: “Drink–Eat–Mobilise” paradigm on the day of surgery (clear fluids within 2 h, oral intake within 4 h, first assisted walk within 6 h)
• Structured physiotherapy beginning on postoperative day 0–1 with functional objectives (sitting, standing, walking with assistance, stairs)
• Multimodal analgesia: paracetamol, NSAID unless contraindicated, patient-controlled oral opioid if pain score > 4/10)
• Early removal (within 24 h) of intravenous lines and urinary catheter (if inserted in case of postoperative urinary retention)
• Venous thrombo-embolism prophylaxis with low molecular weight heparin from 6 h postoperatively, followed by direct oral anticoagulants for 35 days
• Hospital discharge criteria: adequate pain control with standard oral analgesics, independent ambulation with full weight-bearing using two crutches over at least 100 m, independent stair negotiation, and knee flexion ≥ 90° following total knee arthroplasty
• Post-hospitalisation follow-up (nurse, physiotherapist, surgeon

*NSAID* non-steroidal anti-inflammatory drug

### Data collection

Clinical characteristics (age, gender, height, weight, body mass index, pain scores, complications, length of stay and 30-day readmission) were auto-extracted from the hospital data warehouse and cross-checked weekly. Process-of-care data (element-level adherence) were entered using a REDCap™ registry. During the hospital stay, patients were monitored for pain, functional recovery, and overall satisfaction using either a paper or digital logbook. This promoted active patient involvement and empowerment in their care, leading up to a criteria-based discharge. Comprehensive home support and patient education were provided to ensure a clear understanding of medications, wound care, warning signs, and physiotherapy, including self-directed exercises. Indeed, post-discharge follow-up is initially conducted through digital tools and telemedicine, using our institutional CHUV@Home platform, and continues until the first outpatient follow-up visit at six weeks post-surgery. The clinical pathway includes 24/7 access to a chat service with nurses and physician assistants, as well as automated pop-up patient guidance and advice, ensuring continuous support and engagement.

Data for the ERAS cohort were collected prospectively, with variables predefined before ERAS implementation. Information was gathered from the hospital’s electronic medical records and entered into the Research Electronic Data Capture (REDCap) database.

### Outcomes

The primary outcome was hospital length of stay. Secondary outcomes were rest and dynamic pain scores on postoperative day zero, one and two (11-point numeric scale).; rate of postoperative nausea and.

vomiting on postoperative day zero, one and two; rate of mobilisation on postoperative day zero and one; incidence of in-hospital complications (urinary retention, arrythmia, pneumonia, ileus, neurapraxia, deep venous thrombosis, melena, other, fall with dislocation or fracture, death); rate of reoperation; and 30-day readmission rate.

### Statistical analysis

The ERAS and non-ERAS groups were initially compared using standard univariate statistical methods to assess demographic (age, sex, Body Mass Index, ASA score) similarity. Continuous variables are expressed as mean ± standard deviation; categorical variables as counts and percentages. Between-group comparisons employed the Student’s t-test, or Fisher’s exact test as appropriate. Statistical significance was set at α = 0.05. Analyses were performed using Stata® 18.0.

## Results

From December 1 st, 2021, to November 30th, 2022, data from 267 patients (138 THA, 129 TKA) were reviewed and compared with data from 258 patients (128 THA, 130 TKA) collected between December 1 st, 2022, and November 30th, 2023, who underwent the ERAS® program (total number of patients: 525 patients). Table 2 displays the demographic data of these patients. The two cohorts are similar in term of age, sex, Body Mass Index, ASA score. Duration of surgery with the ERAS program was significantly shorter for THA and longer for TKA when compared with before ERAS implementation.

**Table 2:** Patient demographics and clinical characteristics

	THA before ERAS®	THA after ERAS®	*p*-value	TKA before ERAS®	TKA after ERAS®	*p*-value
Sex, *n* (%)			0.90			0.71
Male	69 (50.0%)	63 (49.2%)		61 (47.3%)	58 (44.6%)	
Female	69 (50.0%)	65 (50.8%)		68 (52.7%)	72 (55.4%)	
Age, years	68.9 (12.0)	68.6 (12.0)	0.85	70.9 (10.0)	70.4 (10.4)	0.68
Weight, kg	79.3 (17.5)	77.7 (17.2)	0.47	84.3 (18.0)	85.6 (18.5)	0.57
Height, cm	169.1 (9.0)	169.4 (9.0)	0.77	166.8 (10.1)	168.1 (10.4)	0.33
Body mass index, kg.m^−2^	27.7 (5.5)	27.0 (5.2)	0.32	30.3 (5.8)	30.2 (5.5)	0.94
ASA score, *n* (%)			0.19			0.92
I	6 (4.3%)	12 (9.4%)		4 (3.1%)	5 (3.8%)	
II	76 (55.1%)	73 (57.0%)		74 (57.4%)	76 (58.5%)	
III	56 (40.6%)	43 (33.6%)		51 (39.5%)	49 (37.7%)	
Indication, *n* (%)			0.002			0.50
Osteoarthritis	128 (92.8%)	127 (99.2%)		129 (100.0%)	128 (98.5%)	
Osteonecrosis	10 (7.2%)	0 (0.0%)		0 (0.0%)	0 (0.0%)	
Rheumatoid arthritis	0 (0.0%)	1 (0.8%)		0 (0.0%)	2 (1.5%)	
Type of anaesthesia, *n* (%)			0.87			0.21
General	23 (16.7%)	20 (15.6%)		29 (22.5%)	21 (16.2%)	
Spinal	115 (83.3%)	108 (84.4%)		100 (77.5%)	109 (83.8%)	
Peripheral nerve block, *n* (%)	0	0	-	127 (98.4%)	130 (100.0%)	0.25
Duration of surgery, min	58.8 (23.0)	49.2 (18.5)	< 0.001	75.4 (20.6)	83.9 (28.2)	0.006

Continuous data are presented as means and standard deviations and compared by using Student’s t-tests; categorial data are presented as number of patients (%) and compared by using Fisher’s exact tests

*THA* total hip arthroplasty, *TKA* total knee arthroplasty

**Fig. 1:**
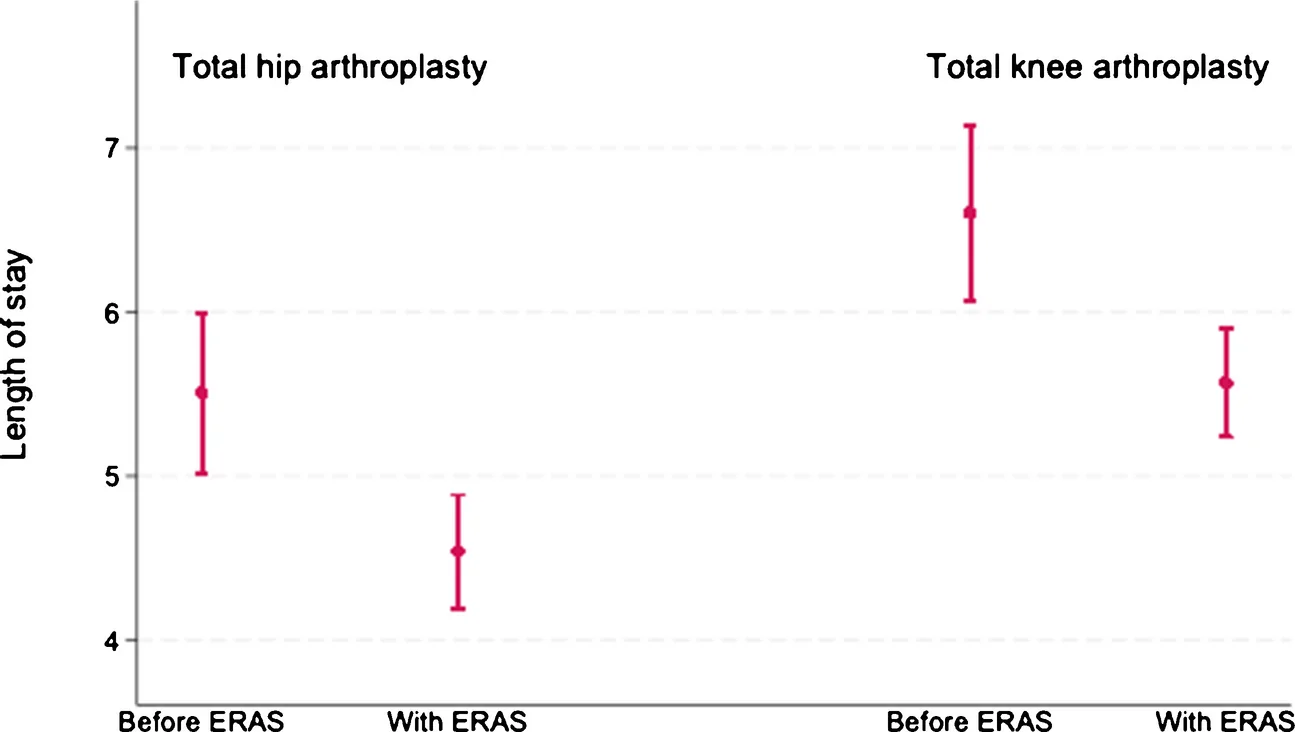
Length of stay

The mean hospital length of stay for patients scheduled for THA before ERAS® was 5.5 ± 2.9 days versus 4.5 ± 2.0 days after ERAS® implementation (*p* = 0.002) (Fig. 1). For patients having a TKA, mean hospital length of stay was 6.6 ± 3.1 days before vs 5.6 ± 1.9 days after ERAS® implementation (*p* = 0.001) (Fig. 1). Rest and dynamic pain scores, and the rate of nausea and vomiting were similar between groups except for rest pain score on POD 0 and dynamic pain score on POD 1 that were both reduced in post-ERAS THA patients (Table 3), but without reaching clinical relevance. Patients undergoing THA were more frequently mobilised with ERAS® on POD 0. Rates of complications were similar between groups except for pneumonia in patients undergoing TKA (Table 4).

**Table 3:** Pain-related secondary outcomes

	THA before ERAS®	THA with ERAS®	*p*-value	TKA before ERAS®	TKA with ERAS®	*p*-value
Rest pain score						
POD 0	1.0 (1.7)	0.5 (1.0)	0.005	0.9 (1.4)	0.7 (1.5)	0.22
POD 1	1.5 (1.7)	1.1 (1.4)	0.06	2.1 (1.8)	2.0 (2.0)	0.68
POD 2	1.4 (1.4)	1.4 (1.1)	0.70	2.3 (1.5)	2.4 (1.6)	0.56
Dynamic pain score						
POD 0	1.1 (1.7)	0.7 (1.4)	0.07	1.3 (1.8)	1.0 (1.8)	0.14
POD 1	2.5 (2.1)	2.0 (1.8)	0.03	3.3 (2.2)	3.1 (2.2)	0.42
POD 2	2.9 (1.5)	2.7 (1.5)	0.26	3.6 (1.7)	4.0 (1.8)	0.07
Nausea and vomiting						
POD 0	5 (3.6%)	4 (3.1%)	0.55	3 (2.3%)	5 (3.8%)	0.37
POD 1	8 (5.8%)	5 (3.9%)	0.58	3 (2.3%)	5 (3.8%)	0.72
POD 2	8 (5.8%)	5 (3.9%)	0.58	6 (4.7%)	8 (6.2%)	0.78
Mobilization	x					
POD 0	51 (37.0%)	67 (52.3%)	0.014	68 (53.1%)	73 (56.2%)	0.71
POD 1	135 (97.8%)	128 (100.0%)	0.25	124 (96.9%)	128 (98.5%)	0.44

Continuous data are presented as means and standard deviations and compared by using Student’s t-tests; categorial data are presented as number of patients (%) and compared by using Fisher’s exact tests. NRS, numeric rating scale: 0 (no pain) to 10 (worst imaginable pain)

*THA* total hip arthroplasty, *TKA* total knee arthroplasty

**Table 4:** Rate of complications

	THA before ERAS®	THA with ERAS®	*p*-value	TKA before ERAS®	TKA with ERAS®	*p*-value
Complications			0.12			0.41
None	120 (87.0%)	120 (93.8%)		114 (88.4%)	121 (93.1%)	
< Grade IIIb	17 (12.3%)	8 (6.2%)		13 (10.1%)	8 (6.2%)	
≥ Grade IIIb	1 (0.7%)	0 (0.0%)		2 (1.6%)	1 (0.8%)	
Complications < Grade IIIb						
Urinary retention	6 (4.3%)	2 (1.6%)	0.28	6 (4.7%)	3 (2.3%)	0.33
Arrhythmia	3 (2.2%)	1 (0.8%)	0.62	0 (0.0%)	1 (0.8%)	1.00
Pneumonia	4 (2.9%)	1 (0.8%)	0.37	5 (3.9%)	0 (0.0%)	0.03
Ileus	0 (0.0%)	2 (1.6%)	0.23	0 (0.0%)	0 (0.0%)	-
Neurapraxia	0 (0.0%)	1 (0.8%)	0.48	1 (0.8%)	0 (0.0%)	0.50
Deep vein thrombosis	0 (0.0%)	0 (0.0%)	-	0 (0.0%)	1 (0.8%)	1.00
Melena	0 (0.0%)	0 (0.0%)	-	1 (0.8%)	1 (0.8%)	1.00
Other	4 (2.9%)	1 (0.8%)	0.37	0 (0.0%)	2 (1.5%)	0.50
Complications ≥ Grade IIIb						
Fall (dislocation, fracture)	0 (0.0%)	0 (0.0%)	-	1 (0.8%)	1 (0.8%)	1.00
Death	1 (0.7%)	0 (0.0%)	1.00	1 (0.8%)	0 (0.0%)	0.50
Reoperation, *n* (%)	1 (0.7%)	2 (1.6%)	0.61	2 (1.6%)	2 (1.5%)	1.00
30-day readmission, *n* (%)	1 (0.7%)	2 (1.6%)	0.61	2 (1.6%)	1 (0.8%)	0.62

Categorial data are presented as number of patients (%) and compared by using Fisher’s exact tests

*THA* total hip arthroplasty, *TKA* total knee arthroplasty

## Discussion

In this two-cohort study, the implementation of an ERAS® program for hip and knee arthroplasty led to a reduced hospital length of stay by one day, bringing it below the national average in Switzerland (5 days for THA; 5.4 days for TKA) without impacting pain outcomes, rates of complications or 30-day readmission rate. Of note, the reduction in rest pain score on POD 0 and dynamic pain score on POD 1 in favour of post-ERAS THA patients does not reach the minimal threshold of clinical significance of 1 unit [7] and might be the result of a type II error. Another benefit of our ERAS® program was the increased rate of mobilization on POD 0. However, we cannot determine the long-term impact of this benefit, since we did not measure functional outcomes at several weeks after hospital discharge. The differences in duration of surgery before and after ERAS implementation (increased in THA patients with an average of 10 min and decreased in TKA patients with an average of 9 min) reflect the practice of a university teaching hospital with many fellows implicated in our program. The main reason behind the absence of reduction in the rates of complications is probably that the ERAS guidelines were already quite aligned with our pre-existing clinical practices.

Implementation of our ERAS® pathway resulted in a statistically and clinically significant reduction in hospital length of stay (LOS) for both THA and TKA—approximately one day shorter compared with the pre-ERAS cohort. These findings are consistent with the robust body of evidence demonstrating that structured ERAS or “fast-track” programs markedly shorten hospitalization after joint arthroplasty without increasing readmission or complication rates [2–5]. *Frassanito *et al*.* described mean LOS of 3.6 to 4.3 days following implementation of a multidisciplinary ERAS protocol for hip and knee arthroplasty, depending on ASA score [2]. *Malviya *et al*.* observed a decrease from six to three days with parallel reductions in mortality and transfusion rates in a series of 4,500 unselected patients [3]. Likewise, *Gulotta *et al*.* reported that a dedicated fast-track clinical pathway achieved a mean LOS of 2.6 days for total hip replacement without compromising safety [4]. Moreover, population-level data from the Danish multicentre fast-track program demonstrated that 91% of unselected arthroplasty patients could be safely discharged in four days or less; longer hospital length of stay was related to advanced age, comorbidities and preoperative need of a mobility aid [5]. Taken together, our results align with these contemporary series, confirming that standardised ERAS implementation yields reproducible reductions in LOS even outside high-volume or highly specialised centres. Although ERAS implementation led to a comparable absolute reduction in length of stay for both THA and TKA, patients undergoing knee arthroplasty consistently required longer hospitalization. This difference is well recognised and likely reflects the greater postoperative pain, inflammation, and functional demands inherent to TKA, particularly the need to regain sufficient knee flexion and quadriceps control to meet discharge criteria. The modest absolute decrease of one day observed in our study likely reflects both the pre-existing efficiency of care and institutional differences in discharge logistics. Nonetheless, such incremental gains remain clinically and economically meaningful, reinforcing that adherence to evidence-based perioperative care and early rehabilitation is the principal determinant of shortened recovery rather than any single technical innovation.

A few words on our protocol of regional anaesthesia and multimodal analgesia deserve to be mentioned. Recent literature consistently shows that spinal anaesthesia provides superior short-term outcomes compared with general anaesthesia, including lower early pain scores [8], reduced perioperative morbidity and mortality [9–11], shorter hospitalisation [9, 10], and decreased overall costs [9, 10]. These advantages have led the ICAROS consensus group to recommend neuraxial techniques for total hip and knee arthroplasty [12], and advise the addition of peripheral nerve blocks to optimise postoperative results [13]. In line with these recommendations, our protocol combines an adductor canal block with a selective tibial nerve block to address both anterior and posterior knee nociception [14]. Although local infiltration analgesia remains a valid alternative [15], the absence of consensus regarding optimal formulation and infiltration strategy [16] has prompted our preference for ultrasound-guided regional blocks. For hip arthroplasty, our prior research established that 100 µg of intrathecal morphine provides excellent analgesia as a sole neuraxial adjunct, obviating the need for supplementary peripheral blocks [17]. Contemporary clinical evidence indicates that this dosage does not increase the risk of sedation, respiratory depression, or hypoxaemia [18, 19], supporting a strategy of ward-based observation rather than mandatory high-dependency monitoring. While the American Society of Anesthesiologists advises structured 24-h monitoring (hourly for the first 12 h, then every two hours until 24 h) [20], the cumulative data suggest that such vigilance may be unnecessarily conservative and contributes to excessive use of hospital resources without improving safety outcomes. Aligned with ERAS principles, perioperative pain management should employ a multimodal, opioid-sparing regimen inclusive of dexamethasone (0.1–0.2 mg kg⁻^1^) [21].

Optimization during surgery is a central tenet of ERAS programs for hip and knee arthroplasty, emphasizing adherence to standardized protocols, minimally invasive approaches, and judicious use of technology. Streamlined and reproducible workflows in the operating theatre reduce variabi lity in outcomes and help patients achieve discharge criteria more efficiently [22, 23]. Although numerous comparative studies have evaluated surgical approaches—such as the direct anterior versus the posterolateral route in total hip arthroplasty—there is no consistent evidence that one confers superior recovery within an ERAS framework [24]. Indeed, large cohort analyses demonstrate that early discharge can be reliably achieved using conventional posterior or medial parapatellar incisions for hip and knee replacements, respectively [25], suggesting that consistency of technique and team coordination may be more impactful than specific procedural choices. The growing preference for minimally invasive surgery without tourniquet or drain use aligns with ERAS principles, as tourniquets have been associated with delayed rehabilitation, greater postoperative pain, and an elevated risk of thrombotic and wound complications, without demonstrable benefits in blood conservation or cement fixation [26, 27]. Similarly, the routine use of surgical drains is increasingly discouraged, since robust evidence shows no reduction in haematoma or infection rates, and possible increases in bleeding and transfusion requirements—contradicting ERAS objectives of minimal invasiveness and rapid recovery [28, 29]. Successful omission of drains in established ERAS protocols without adverse outcomes further supports this shift in practice [30]. Robotic-assisted techniques offer potential for improved accuracy and tissue preservation, yet they have not independently demonstrated accelerated recovery or discharge. That said, other devices have been developed to improve postoperative outcome in the setting of ERAS [31] Overall, intraoperative performance within ERAS such as duration of surgery [32] depends less on any single innovation and more on consistent, team-based adherence to a standardized, minimally invasive, and time-efficient operative process.

The next evolution of ERAS will likely centre on individualised pathways that adapt to patient-specific characteristics. Predictive analytics and digital patient profiling may allow precision tailoring of perioperative interventions, enable more efficient use of resources and potentially improving outcomes. Emerging technologies—including robot-assisted procedures, wearable monitoring systems, and remote rehabilitation platforms—are poised to enhance postoperative functional recovery and further reduce hospital length of stay. Moreover, these developments could support the wider adoption of same-day or short-stay arthroplasty [33–35], as contemporary data indicate that ambulatory surgery achieves complication rates equivalent to those of inpatient care [36, 37]. Building on successful implementation in primary hip and knee replacement, ongoing work aims to extend ERAS principles to revision arthroplasty, shoulder replacement, and soft-tissue reconstruction such as rotator cuff repair.

Several limitations should be acknowledged. This work reflects a single-institution experience, which may limit the external validity of our findings given potential differences in resources, infrastructure, and patient demographics across centres. Furthermore, the observation period was relatively short; therefore, longer-term durability of compliance and outcome improvement remains to be determined. Although perioperative adherence and selected clinical indicators were measured, patient-centred outcomes—such as health-related quality of life, satisfaction, or longer-term functional recovery—were not evaluated. Future multicentre studies incorporating these dimensions are needed to confirm and extend the present findings on ERAS implementation in orthopaedic surgery.

## Conclusion

In conclusion, the implementation of an ERAS® program for hip and knee arthroplasty led to a reduced hospital length of stay, below the Swiss national average, without impacting pain outcomes, rates of complications and 30-day re-admission rate.

## Data Availability

Data are available upon reasonable request sent to the corresponding author.
